# Low-Temperature Ammonia Sensing Using Sulfurized WS_2_ Thin Films

**DOI:** 10.3390/s26144429

**Published:** 2026-07-12

**Authors:** Naman Kumar, Ivan Hotový, Magdaléna Kadlečíková, Michaela Sojková, Ivan Kostič, Dagmar Gregušová, Ondrej Pohorelec, Viera Stopjaková, Edmund Dobročka

**Affiliations:** 1 Faculty of Electrical Engineering and Information Technology, Slovak University of Technology, Ilkovičova 3, 81219 Bratislava, Slovakia; naman.kumar@stuba.sk (N.K.); magdalena.kadlecikova@stuba.sk (M.K.); viera.stopjakova@stuba.sk (V.S.); 2 Institute of Informatics, Slovak Academy of Sciences, Dúbravská cesta 9, 84507 Bratislava, Slovakia; ivan.kostic@savba.sk; 3 Institute of Electrical Engineering, Slovak Academy of Sciences, Dúbravská cesta 9, 84104 Bratislava, Slovakia; michaela.sojkova@savba.sk (M.S.); elekgreg@savba.sk (D.G.); ondrej.pohorelec@savba.sk (O.P.); edmund.dobrocka@savba.sk (E.D.)

**Keywords:** tungsten disulfide (WS_2_), magnetron sputtering, sulfurization, NH_3_ gas sensing, gas response and sensitivity

## Abstract

This study addresses key challenges in gas sensing, particularly achieving high sensitivity at low operating temperatures. Tungsten disulfide (WS_2_) thin films were deposited by sputtering and sulfurized at 800 °C for 30 and 60 min on quartz and sapphire substrates. Raman spectroscopy confirmed multilayer WS_2_ formation, with characteristic modes at 349 and 417 cm^−1^. X-ray diffraction revealed that the films are polycrystalline with the formation of the hexagonal 2H-WS_2_ phase on both substrates. FESEM images showed clearly visible nanoparticles, but they were not evenly distributed and were differently oriented, with a hint of texturing. The WS_2_ films exhibited stable sensing performance at low operating temperatures from 30 to 150 °C and high sensitivity toward NH_3_ in the 10–200 ppm range. Experimental results indicate a limit of detection, highlighting the potential of WS_2_ films for low operating temperature ammonia sensing. A minimum value of the limit of detection of 0.6 ppm for an operating temperature of 150 °C was achieved for the WS_2_ sample sulfurized at 800 °C for 60 min on a quartz substrate.

## 1. Introduction

In recent decades, metal oxide semiconductors (MOXs) have dominated gas-sensing applications due to their low cost, fast response and recovery, and high sensitivity at elevated temperatures. However, their major limitation is the requirement for high operating temperatures, resulting in significant power consumption [1]. Most commercial metal oxide semiconductor gas sensors require a continuous power supply in the range of ~66–900 mW to maintain optimal performance [2,3,4,5]. These limitations hinder their use in low-power and portable sensing devices. An ideal gas sensor should combine low fabrication and operating costs, low power consumption, high reliability, repeatability, and real-time response. Emerging low-dimensional materials (0D, 1D, and 2D), including quantum dots, nanowires, nanotubes, and nanosheets, offer strong potential for low-power sensing [6]. However, materials such as polymers, phosphorene, and carbon-based nanostructures often suffer from poor selectivity, limited long-term stability, and signal drift despite their room-temperature operation [7]. Transition metal dichalcogenides, such as MoS_2_ and WS_2_, are layered materials with strong in-plane covalent bonding and weak van der Waals interlayer interactions, making them attractive for gas-sensing applications [8,9,10,11]. Among these, WS_2_ exhibits promising properties for ammonia detection. Theoretically, WS_2_ shows strong adsorption toward gases such as NH_3_, O_2_, and CO, and experimental studies report higher NH_3_ sensitivity compared to MoS_2_ [12,13,14]. In addition, WS_2_ demonstrates ambipolar transport behavior, high thermal conductivity (~142 W m^−1^ K^−1^) [15,16], and high carrier mobility (>1000 cm^2^ V^−1^ s^−1^) [17,18,19,20], along with a direct band gap of ~2.1 eV and an indirect band gap of ~1.3 eV [21,22]. Ammonia (NH_3_) is a toxic and corrosive gas widely used in the agriculture industry [23], and it also serves as a biomarker for several diseases. Therefore, the development of sensitive NH_3_ sensors operating at room temperature and low concentrations is of significant importance, and boosts user-friendliness [24,25,26,27,28,29,30].

As reported by Hau et al. (2026) [31], WS_2_ exhibited the highest selectivity toward NO_2_, whereas NH_3_ was the second most responsive analyte; although H_2_S and SO_2_ produced measurable cross-responses, H_2_ and CO induced negligible signals, indicating limited sensitivity to these gases. Similarly, Sharma et al. (2023) [32] showed that WS_2_ was most selective toward NH_3_, followed by formaldehyde, while ethanol and acetone elicited only weak cross-responses and benzene produced a negligible response. More broadly, the WS_2_ literature has most frequently emphasized selective sensing toward two gases, NO_2_ and NH_3_, representing oxidizing and reducing species, respectively. Since these analytes typically drive conductance in opposite directions depending on the intrinsic conduction type of WS_2_, cross-selectivity between them is not typically anticipated.

WS_2_ nanostructures, particularly those with high surface-to-volume ratios, enable efficient gas adsorption even at room temperature. Magnetron sputtering, a physical vapor deposition technique, addresses key fabrication limitations by enabling the deposition of dense, uniform, and highly crystalline thin films with precise nanoscale thickness control, strong substrate adhesion without post-deposition treatment, and reduced particle aggregation [33,34,35,36]. However, sputtered WS_2_ films often suffer from sulfur deficiency due to preferential re-sputtering and weak sulfur bonding. Post-deposition sulfurization is therefore essential to restore stoichiometry and improve sensing performance [37]. This process can also promote the formation of vertically oriented, edge-rich WS_2_ structures, which provide a high density of active sites and enhance gas response. The results achieved with WS_2_-based sensors in other laboratories are noteworthy. O’Brien et al. (2014) report thin WS_2_ films (2 to 50 nm) synthesized from WO_3_ by plasma-assisted H_2_S sulfurization, which show responses of 0.005% to 0.2% for 1–200 ppm NH_3_ at room temperature, with response times of a few seconds, but with poor recovery [38]. Some WS_2_ sensors achieve a detection limit of 1.4 ppm for ammonia [38]. Järvinen et al. (2019) report WS_2_ thin films (~20 nm thick) on SiO_2_/Si substrates prepared by tungsten sputtering followed by sulfurization with Ti/Pt electrodes, achieving ~33% response to 1–10 ppm NH_3_ at room temperature, response time ~5 min, recovery time ~6 min, and sensitivity 0.10 ± 0.02 ppm^−1^ [14]. Fedorenko et al. (2025) described WS_2_ thin films (~30 nm thick) grown on SiO_2_/Si substrates by magnetron sputtering of tungsten followed by sulfurization at 1000 °C in an H_2_-containing atmosphere, although the response to 100 ppm NH_3_ at room temperature is ~2.8% [39].

In this study, WS_2_ thin films are deposited by magnetron sputtering on quartz and sapphire substrates at room temperature and subsequently sulfurized at 800 °C for 30 and 60 min. The structural and morphological properties of the films are correlated with their gas-sensing performance. The influence of substrate type and sulfurization duration on gas response and sensitivity is analyzed, demonstrating the potential of WS_2_ films for efficient low operating temperature ammonia sensing.

## 2. Materials and Methods

### 2.1. Preparation of WS_2_ Films

The preparation of WS_2_ films consists of the following steps. The substrate material is quartz glass and synthetic sapphire. The plane of sapphire on which the films were deposited has a crystallographic orientation of c-plane [0001] (Cryscore optoelectronics Ltd., Jiaozuo, China). High-purity WS_2_ composite target material (99.9% purity, fy MaTeck, Juelich, Germany) was used for deposition onto unheated polished 10 × 10 mm^2^ quartz and sapphire substrates. Thin WS_2_ films were fabricated by high-vacuum direct-current magnetron sputtering in an argon atmosphere at a working pressure of 0.6 Pa, using a sputtering power of 45 W and a target to substrate distance of 75 mm. Before deposition, the quartz and sapphire substrates were ultrasonically cleaned sequentially in acetone, isopropyl alcohol, and deionized water to remove surface contaminants. The WS_2_ thin films had a thickness of 28 nm after sputtering, measured by the Talystep profilometer. The second technological step was sulfurization. During this step, WS_2_ films on different substrates were placed into a quartz crucible in the center of the tube furnace together with the sulfur powder (1.0 g of Sulphur) and heated at a rate of 25 °C/min to the process temperature. Temperature and annealing times were 800 °C, 30, and 60 min, respectively, which was optimized based on our research [40,41,42,43]. The sulfurization was performed in a nitrogen atmosphere at ambient pressure. After sulfurization, the temperature was decreased at a rate of 20 °C/min down to 200 °C, followed by spontaneous cooling. Sulfurization parameters as well as identification of the samples examined are listed in Table 1.

### 2.2. WS_2_ Characterization Techniques

The surface morphologies of the prepared WS_2_ films were observed by field emission scanning electron microscopes (FESEM, JSM 7500F, JEOL, Tokyo, Japan, and FESEM, Quanta FEG 250, FEI Company, Hillsboro, OR, USA) in secondary electron mode and combined secondary and backscattered electron mode. To analyze the results obtained from FESEM, we employed the open-source software package, ImageJ v1.54, to evaluate various objective parameters present in the sample, including particle size and density. Raman spectroscopy was carried out in a Jobin Yvon Spex Labram confocal Raman spectrometer (ISA Dilor-Jobin YvonSpex Labram, HORIBA Group, Lyon, France) with a 632.8 nm He-Ne laser in a backscattering configuration, a laser power of 5 mW, and spectral resolution better than 1.3 cm^−1^. Microscope objectives 10× and 80× were used to focus the laser beam onto a spot of approximately 20 µm in diameter, and to collect the scattered light, which then passed through the spectrometer onto a CCD detector. In addition, a confocal hole with a diameter of 600 µm, a spectrograph entrance slit of 600 µm, and a 600 grooves/mm diffraction grating were employed. Calibration measurements were performed on single crystalline Si (100) with the catalogue value for silicon 520.7 cm^−1^. All Raman spectra were recorded at room temperature. At least three Raman spectra were measured on individual samples at different locations diagonally across each sample to confirm uniform WS_2_ coverage. The spectral window was from 250 to 480 cm^−1^. For structural analysis, a Bruker D8 DISCOVER diffractometer was used. This was equipped with an X-ray source with a rotating Cu anode (λ = 0.15418 nm) operating at 12 kW (40 kV/300 mA). All measurements were performed in a parallel beam geometry with a parabolic Goebel mirror in the primary beam. The X-ray diffraction patterns were recorded in the 2θ range of 10–40° with a step size of 0.05°. Sheet resistance measurements were carried out using a four-point probe system (Ossila) to minimize contact resistance effects and obtain reliable sheet resistance values; the four collinear probes were brought into gentle contact with the film surface, with a known current passed through the outer probes and the corresponding voltage drop recorded across the inner probes. The temperature variation in conductance of the prepared WS_2_ films was measured by Au tips in a special LINKAM temperature-controlled chamber and an Agilent multimeter in the range of 60 °C to 200 °C. The resulting data were fitted to an Arrhenius plot to extract the activation energy for electrical conduction. From the Arrhenius equation, we know, σ=σ0e−EaKT, where T is temperature, K is the Boltzmann constant, E_a_ is the activation energy, σ is the conductivity dependent on temperature, and $\sigma_{0}\;$ is a constant. To test the sensing properties of the prepared WS_2_ layers, the LINKAM chamber was used to heat the samples and control the gaseous atmosphere, while electrical measurements were performed using Au tips. The gas mixing system, consisting of a mass flow system connected to different gas flow controllers (Red-y) and a gas transfer system of stainless steel pipelines and valves, can prepare a complex gas mixture. Certified bottles of NH_3_ (1000 ppm in Ar) and carrier gas nitrogen were used for the NH_3_ sensing experiments. The gas mixing was regulated by varying the gas flows from the ammonia and nitrogen bottles through the mass flow controllers to adjust the NH_3_ concentration in the range of 10–200 ppm in nitrogen.

**Figure 1:**
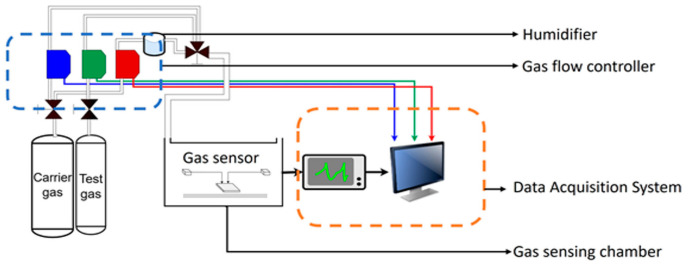
Schematic diagram of gas sensing measurement setup.

During the measurements, a constant gas flow (200 sccm) through the measurement chamber was maintained, and a constant relative humidity of 60% was measured and maintained. The sensor working temperature was controlled and varied from near room temperature (30 °C) to 150 °C. The response resistance was measured by an Agilent multimeter and LabVIEW (National Instruments) as a data acquisition setup (see Figure 1). The gas response S is defined as Equation (1)

(1) $$S\left(\%\right)=\frac{R_{g}-R_{0}}{R_{0}},$$

where R_g_ is the resistance value at the end of the exposure time to NH_3_, and R_0_ is the resistance of the baseline in air. The gas mixing test bench is completely automatic; a personal computer runs software that controls the operations of data acquisition, storing and plotting in real time the dynamic response curve of the examined sample. To assess the minimum NH_3_ concentration that our samples are capable of detecting, the limit of detection (LOD parameter) was defined as Equation (2)

(2) LODppm=3σnoiseM

where M is the slope of the calibration curve, response [%] versus concentration [ppm], and $\sigma_{noise}$ is the standard deviation of residual noise.

## 3. Results

### 3.1. Morphology of WS_2_

**Figure 2:**
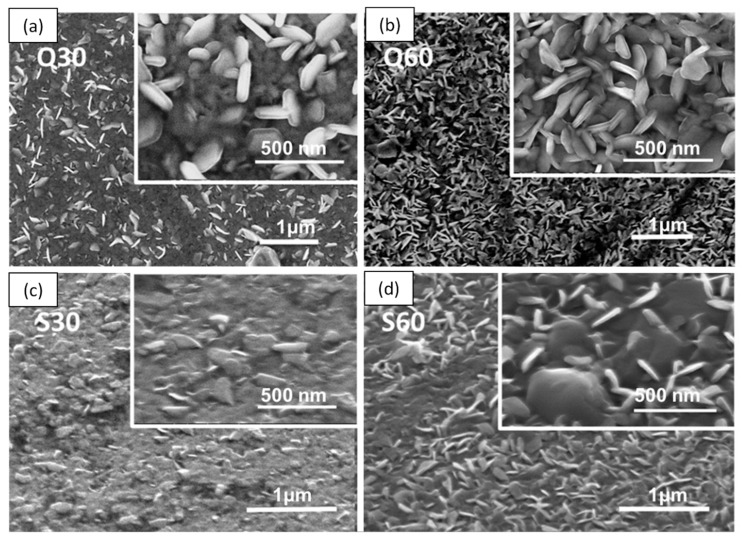
FESEM images depicting the morphology of WS_2_ (**a**,**b**) films on quartz substrates and (**c**,**d**) films on sapphire substrates.

Figure 2 shows the morphology of nanostructured films on WS_2_ grown on quartz and sapphire substrates. FESEM characterizes the distribution of the crystal seed layer, growing crystals, and nanoflakes, which are clearly visible. The WS_2_ flakes are not evenly distributed and are differently oriented. This is related to the fact that WS_2_ crystals have strong in-plane chemical bonds but weak interplanar bonds, so they can form nanoflakes or nanolayers that are nanometers thick in one direction but also micrometers wide. Comparing Figure 2a,b, it is clear that a longer sulfurization time, 60 min, leads to a more pronounced development of WS_2_ flakes. Comparing the morphology of WS_2_ layers on the quartz substrate (Figure 2a,b) versus WS_2_ layers on the sapphire substrate (Figure 2c,d), it is clear that crystallization on the sapphire substrate proceeds more slowly than on the quartz substrate. After 30 min of sulfurization, a WS_2_ layer on sapphire (see Figure 2c), a crystal seed layer, is evident, which is continuous on the surface substrate and growing crystals, and after 60 min of sulfurization, already developed nanoflakes are visible (see Figure 2d). We believe that the strength of the crystallization is related to the thermal conductivity of the substrates. While quartz glass is almost an insulator, sapphire has a high thermal conductivity (up to 40 times greater than glass). Therefore, the heat required for crystal growth is conducted to the substrate holder [44]. The temperature required for WS_2_ crystallization on a quartz substrate is approximately 200 °C.

### 3.2. The Percentage of Image Area Covered by Nanoparticles

**Figure 3:**
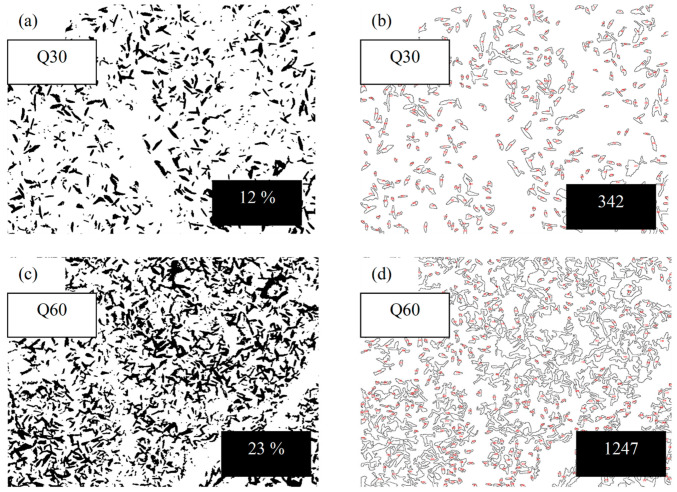
Binary image of the nanostructure WS_2_ on quartz substrates, (**a**,**c**) percentage of the image area covered by all particles, (**b**,**d**) total number of detected particles.

The number of nanoparticles per analyzed area is significantly higher in samples Q60 and S60 than in samples Q30 and S30, in connection with the sulfurization period. It turns out that the alternative substrate fundamentally affects the crystallinity of the WS_2_ layer due to the different surface and lattice parameters of the materials used. Figure 3 shows a binary image of the nanostructure WS_2_ on quartz substrates, with the percentage of the image area covered by all particles and the total number of detected particles. The calculated average WS_2_ flake thickness for the films is given in Table 2. The synthetic sapphire substrate is crystalline and allows for the so-called van der Waals epitaxy. It supports the growth of layers with the basal plane parallel to the substrate (horizontal orientation), which leads to the formation of a continuous layer. The higher binding energy at the WS_2_/sapphire interface compared to amorphous substrates supports lateral growth, and larger crystalline domains are visible (see image of sample S30 in Figure 2c). In this case of amorphous quartz, the deposited layers tend to form randomly oriented domains or vertical domains (nanoflakes) with a high proportion of grain boundaries. Our experiment showed that a higher density of smaller WS_2_ nanoflakes is formed on quartz compared to the sapphire substrate. The preferred tendency for vertical growth of nanostructures on amorphous substrates increases the roughness and active area of the WS_2_ layer. The average thickness of the WS_2_ nanoflake is ∼20 nm.

### 3.3. Raman Characteristics of WS_2_ Films

**Figure 4:**
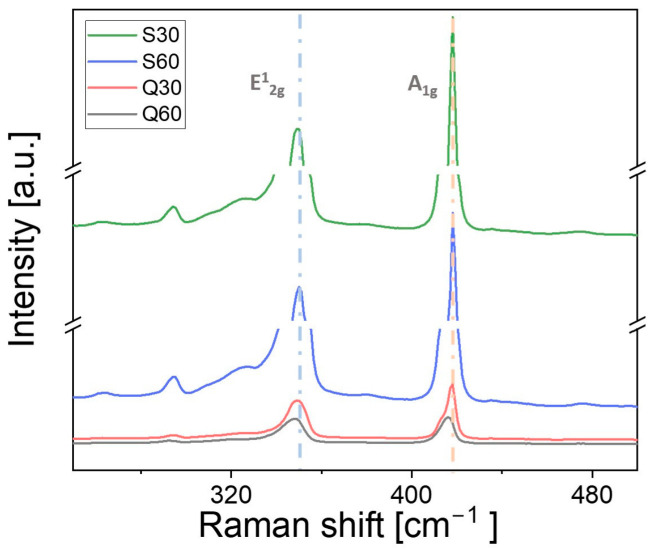
Raman spectra of the dominant peaks of WS_2_ films deposited on quartz and sapphire substrates under He-Ne laser excitation.

In the graphically recorded Raman characteristics of WS_2_ samples in Figure 4, two bands with centers at 346 cm^−1^ to 350 cm^−1^ are dominant, which approximately correspond to the E^1^_2g_ mode (in-plane vibrations of W and S atoms) and a band with a center at approximately 418 cm^−1^, which corresponds to the A_1g_ mode (out-of-plane vibrations of S atoms). The second-order longitudinal acoustic mode 2LA in the Raman spectrum of WS_2,_ excited by a He-Ne laser, may be present, overlapped, absent, or significantly attenuated near the E^1^_2g_ band. To ensure the detection of the 2LA mode, it is important to optimize the experimental conditions. We did not analyze the 2LA mode in the samples studied. An estimate of its position from the deconvolution process is approximately 344 cm^−1^, and is also present due to longitudinal acoustic phonons at the M point of the Brillouin zone. The spectra exhibited identical features for WS_2_ on quartz and on sapphire substrates, differing only in their intensities. The intensity of the Raman response of the WS_2_ layer on sapphire is approximately 10 times higher than that of the WS_2_ layer on quartz.

Table 3 describes the parameters of Raman characteristics derived from the deconvolution of Raman spectra. The difference between the recorded peaks of the dominant bands of the samples under study is approximately 67 cm^−1^. The intensity ratio A_1g_/E^1^_2g_ of the Raman peaks varies depending on the samples. The decrease in the intensity of the E^1^_2g_ mode may be related to defects, mainly sulfur vacancies, or amorphous regions in the WS_2_ layers, where certain vibrations are not effectively scattered. In multilayer WS_2_, the layers are bound to each other by van der Waals forces, and these interlayer interactions can change the dynamics of vibrations, which also leads to the attenuation of the E^1^_2g_ mode. The FWHM of both dominant modes of an ideal WS_2_ crystal or monolayer is 3 to 6 cm^−1^. The Raman peaks of crystalline semiconductors are sharp because of the well-defined wave vectors of the phonons. Defects disrupt the periodicity of the crystal lattice, which leads to a decrease in phonon coherence and therefore to a slight broadening of the full width at half maximum of both characteristic bands of E^1^_2g_ and A_1g_. The FWHM of the A_1g_ mode of all samples is smaller than the FWHM of the E^1^_2g_ mode of all samples. Fewer defects on the ordered surface of the sapphire substrate also led to a better crystallographic quality of the WS_2_ layer and bandwidth reduction. The calculated FWHM values show a clear correlation between the crystallographic (morphological) quality of WS_2_ and the observed Raman response intensity. The larger FWHM of the E mode for WS_2_/quartz is a consequence of the defect density or the amorphous nature of the substrate. The narrower FWHM values for WS_2_/sapphire at the E mode (plane vibrations) are directly related to the lower degree of local mechanical stress and better layer homogeneity. The amorphous surface of quartz certainly has a higher defect density (than the crystalline surface of sapphire), which undesirably shortens the time of scattering centers (phonons and excitons). This leads to a broadening of the FWHM and the Raman spectrum of WS_2_/quartz. The dominant factor explaining the 10x higher Raman scattering intensity in the WS_2_ layer deposited on sapphire is the higher refractive index of the sapphire substrate compared to quartz. The higher refractive index of sapphire leads to stronger constructive interference of both incident and scattered light within the layer, which amplifies the local electromagnetic field and thus the measured signal. A higher refractive index of sapphire means that the WS_2_/sapphire interface reflects light into the layer with much greater efficiency than the WS_2_/quartz interface.

### 3.4. XRD Results

**Figure 5:**
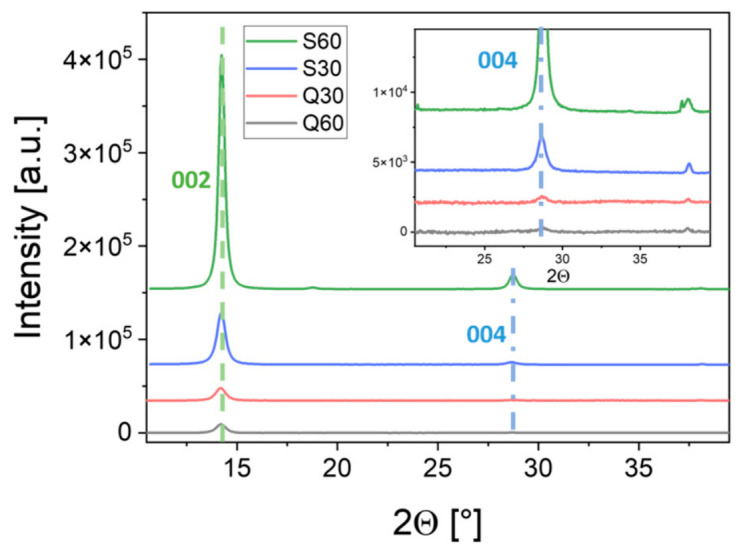
XRD diffractogram of WS_2_ films deposited on quartz and sapphire substrates with peak positions from WS_2_-PDF-08237.

Figure 5 shows the XRD diffractogram recorded on the WS_2_ films in the range of 2θ from 10 to 40 degrees under parallel beam geometry. The diffraction patterns of the sulfurized films confirm the formation of the hexagonal 2H-WS_2_ phase on both substrates, with no detectable secondary phases, indicating that post-deposition sulfurization effectively restores a near-stoichiometric WS_2_ composition. The dominant reflection at 2θ ≈ 14° is indexed to the 002 diffraction planes, indicative of a preferential out-of-plane orientation of the WS_2_ layers parallel to the substrate surface. For films deposited on quartz, prominent peaks corresponding to the WS_2_ 002 and 004 crystalline planes emerged progressively, confirming improved crystallinity with prolonged deposition. 002 and 004 are diffraction indices; they describe the 2nd and 4th order diffractions from the same lattice planes (001). The 002 reflection became sharper and more intense with increasing sulfurization duration, consistent with enhanced long-range ordering and an increased average crystallite size as determined by Scherrer analysis. Structural parameters of WS_2_ films from XRD analysis and the deviation from our recorded positions to the standard peak position of WS_2_-PDF-080237 are listed in Table 4. Irrespective of sulfurization time, all deposited WS_2_ films were polycrystalline. The average crystallite size was determined using the Debye–Scherrer equation, Equation (3) [11].

(3) $$D=\frac{0.89\lambda }{\beta cos\theta },$$

D is the average crystallite size, and λ is the K-alpha doublet X-ray wavelength (λ = 0.15418 nm). Here, β and θ are the FWHM and Bragg diffraction angle of the selected reflection, respectively. The most intense 002 reflection was used to estimate crystallite size for films on quartz.

### 3.5. Electrical Properties

**Figure 6:**
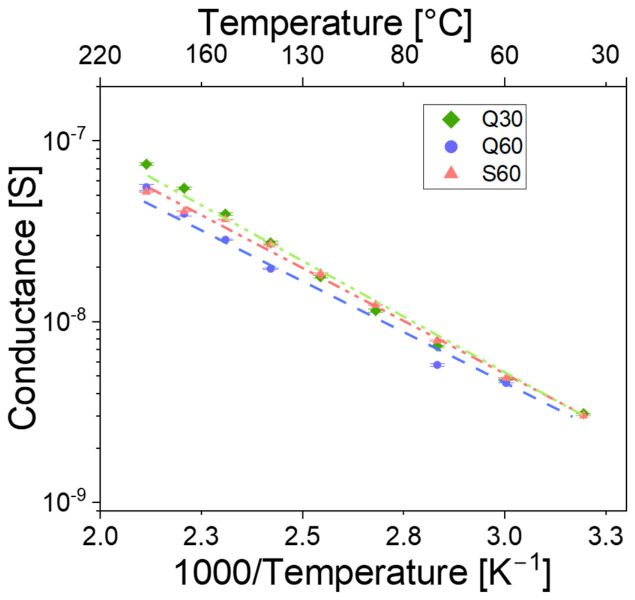
Arrhenius plots of the electrical conductivity of the WS_2_ layers.

The values of sheet resistance as well as the temperature dependence of the resistance of the prepared WS_2_ films, are suitable parameters that characterize the suitability of the material for use in gas microsensors and also reveal the electrical nature of the investigated material. For WS_2_ films Q30 and Q60, the sheet resistance of 5 MΩ/□ and 18 MΩ/□ was measured using the four-probe method, which is due to the amount of clearly developed nanoflakes/nanolayers in the case of longer sulfurization time (see Figure 2a,b) and thus also longer paths for the flowing current in the sheet resistance measurement. The measured sheet resistance value for sample S60 was 374 MΩ/□, but for sample S30, the sheet resistance could not be measured due to inhomogeneity and very high resistance. The answer to the recorded increased value can also be found in the observed morphology, which is influenced by the type of substrate. The temperature variation in the conductance in air was measured between 40 and 200 °C. Figure 6 shows a representative temperature dependence of the conductance for the examined WS_2_ films. It is seen that film conductance increases by almost two orders of magnitude between 40 and 200 °C, and all samples were p-type semiconductors in the examined temperature range. We observed a strong exponential dependence, which may be due to thermal excitation of carriers into the conduction band.

### 3.6. Gas Sensing Properties

**Figure 7:**
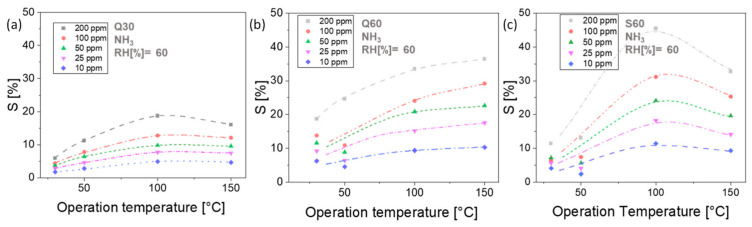
Gas sensing response versus operating temperature (**a**,**b**) for WS_2_ on quartz and (**c**) for WS_2_ on sapphire substrates.

To evaluate the effects of process parameters on the gas-sensing properties of the WS_2_ films, different gas-sensing tests toward NH_3_ were carried out on three samples. The examined WS_2_ films were operated at a constant temperature and exposed to different low NH_3_ concentrations (10, 25, 50, 100, and 200 ppm); after each NH_3_ exposure, a recovery period in humid air followed. The above gas-sequence protocol was repeated in humid air (60% relative humidity) at working temperatures ranging from 30 to 150 °C. The NH_3_ response *versus* operating temperature is shown in Figure 7 for the monitored samples. As is evident from this figure, the gas response towards NH_3_ has a maximum value at a temperature of approximately 100 °C for all samples.

**Figure 8:**
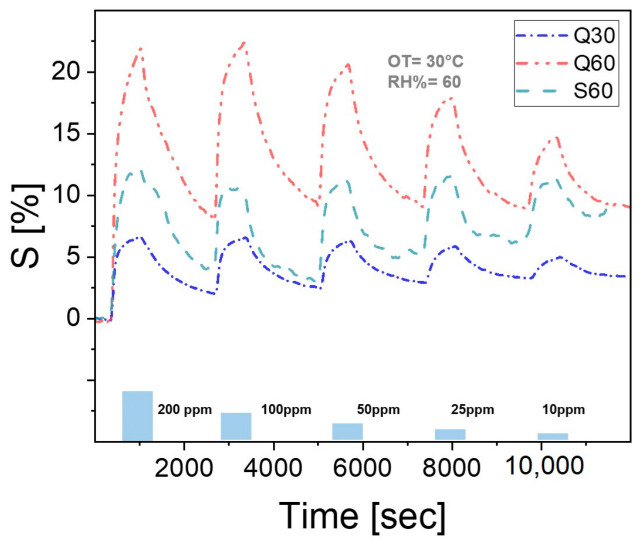
Dynamic response characteristics of the gas sensing of NH_3_ at 30 °C.

**Figure 9:**
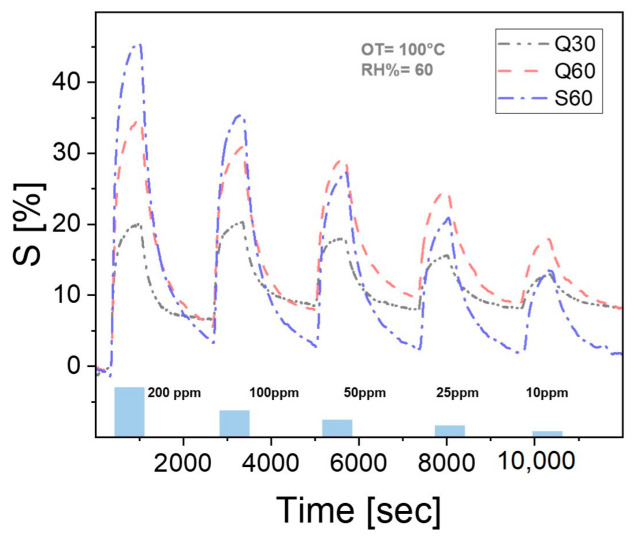
Dynamic response characteristics of the gas sensing of NH_3_ at an operating temperature of 100 °C.

**Figure 10:**
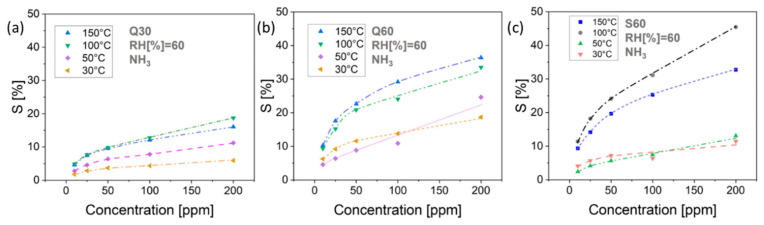
Calibration curve as a function of NH_3_ concentration at temperatures 30 °C up to 150 °C, (**a**,**b**) for WS_2_ on quartz and (**c**) for WS_2_ on sapphire substrates.

**Figure 11:**
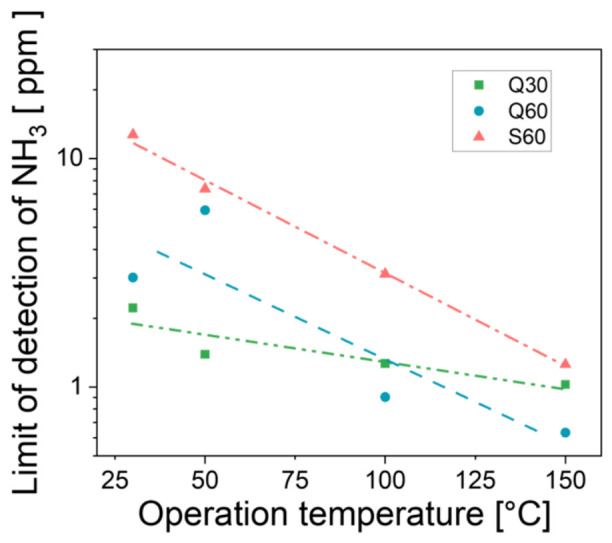
Limit of detection of NH_3_ of WS_2_ films.

Moreover, we can observe that the WS_2_ films were prepared at different process conditions and characterized by different physical properties, but at the same time, they show slightly different responses to NH_3_. Only sample Q60 shows an improvement in gas response even at 150 °C, but the improvement is only slight when comparing temperatures from room temperature to 100 °C. The samples Q30 and S60 have the highest gas responses compared to the Q60 at 100 °C for all investigated NH_3_ concentrations. It is evident from Figure 8 that the ability of all investigated WS_2_ films to detect NH_3_ even at 30 °C is remarkable, with this ability being best seen in the Q60 sample (gas response from 1.7 to 24) with the highest sensitivity. Figure 9 shows the dynamic response of all the WS_2_ films to the NH_3_ measurement protocol at the optimum operating temperature (100 °C) in humid air (60% relative humidity). The sensing signals cannot reach equilibrium during the exposure time, so the response time at this temperature is very low. Sample S60 shows the highest gas response values (from 11.5 to 45.5) for the investigated concentration range at the optimal temperature of 100 °C. Sample Q60 has a similar character (S from 9.5 to 33.5) but with lower sensitivity. Sample Q30 is characterized by low gas response values (from 5 to 18.7) as well as almost half the sensitivity compared to samples S60 and Q60. The calibration curves for ammonia in air at working temperatures from 30 to 150 °C are plotted in Figure 10. In terms of optimal NH_3_ concentrations, for all examined samples, there appears to be an interval of 50 to 200 ppm, in which gas response increases proportionally to the increasing NH_3_ concentration. The S60 film is more sensitive than the Q60, and the WS_2_ film S60 can detect the lowest concentration of NH_3_. Samples S60 and Q60 are more sensitive than sample S30, however, which has a lower detection limit than S60. The LOD values obtained from the lower linear calibration region for Q30 min at RT, 50, 100, and 150 °C were 2.2, 1.4, 1.2, and 1.0 ppm, respectively. In the lower calibration region for Q60, LOD values calculated at RT, 50, 100, and 150 °C were 3.0, 5.9, 0.9, and 0.6 ppm, respectively. Similarly, the LOD values for the S60 WS_2_ film at RT, 50, 100, and 150 °C were determined to be 12.7, 7.3, 3.1, and 1.2 ppm. Among these, the lowest LOD value was identified for Q60 at 150 °C, with a value of 0.63 ppm, indicating the best detection performance at 150 °C (see Figure 11).

## 4. Discussion

Since the structural properties examined by Raman spectroscopy and XRD methods differ only slightly between the individual samples studied, the decisive information about the structure of the films’ influence on the gas sensing properties is provided by the observation in the SEM. Therefore, we will search for mutual links between surface morphology and gas response to NH_3_ for both types of studied substrates, as well as the duration of WS_2_ sulfurization. Indeed, FESEM observation revealed significant differences in surface morphology depending on the sulfurization duration (30 and 60 min) and substrate type (quartz and sapphire). Differences in morphology (grain shape, flake density, and size) between WS_2_ layers grown on quartz and sapphire substrates are directly related to their differing thermal conductivities and thermal expansion characteristics. However, the primary causes of these morphological differences lie in the crystalline structure of sapphire versus the amorphous nature of quartz. Sapphire is single-crystalline with hexagonal surface symmetry. This enables so-called epitaxial growth, leading to the formation of well-defined triangular domains [45]. Quartz is an amorphous material; it cannot provide a structural template for the WS_2_ lattice, resulting in random nucleation and chaotic grain orientation, accompanied by a higher density of defects and grain boundaries [46]. For quartz-based samples, the 30 min sulfurization resulted in a relatively smooth and homogeneous surface with fine, uniformly distributed features. In contrast, the 60 min sulfurization led to a more developed surface morphology characterized by increased roughness, a higher density of irregularities, and the presence of microstructural defects, indicating an increase in the effective surface area. Upon detailed analysis, it is clear that the density of vertically oriented flakes after 60 min of sulfurization is greater than the density of the WS_2_ film after 30 min of sulfurization, which may cause a change in conductivity not only due to the internal anisotropic morphology, but also due to changes in barriers at the flake junctions, defects at the edges, and depletion of charge carriers [32,47]. Sapphire-based samples exhibited a more pronounced structural evolution. While the 30 min sulfurization produced a moderately heterogeneous surface, the 60 min sulfurization resulted in a highly developed, anisotropic morphology with larger aggregates and distinct texturing, suggesting a significantly increased specific surface area. The gas response is directly related to the interaction between NH_3_ molecules and the active surface sites of WS_2_ material, which are strongly influenced by surface morphology. The results demonstrate a clear relationship between SEM observed surface features and gas-sensing performance. Samples with increased surface roughness and higher specific surface area (samples Q60 and S60) exhibited significantly enhanced gas response. This behavior can be attributed to the higher density of active adsorption sites available for NH_3_ molecules. In contrast, smoother and more homogeneous surfaces (sample Q30) showed lower gas response due to the reduced number of active interaction sites. Sample S60, sulfurized for 60 min, showed the highest response values, which can be explained by its highly developed and anisotropic surface morphology. However, this structural complexity may also contribute to less stable sensing behavior due to non-uniform adsorption processes.

### 4.1. Effect of Operating Temperature

The gas response exhibited a strong dependence on operating temperature. At lower temperatures (30–60 °C), the gas response was limited due to insufficient activation energy for adsorption processes. In the intermediate temperature range (60–120 °C), the response reached its maximum, corresponding to optimal adsorption–desorption dynamics of NH_3_ molecules on the sensor surface. The optimal operating temperature was observed near 100 °C, signified by combined optimal properties such as low noise, high response, and high sensitivity regardless of substrates, under similar sulfurization conditions for homogeneous WS_2_ films. At higher temperatures (120–150 °C), a decrease in gas response was observed, likely due to accelerated desorption rates, which reduce the residence time of NH_3_ molecules on the surface. According to the authors of [48,49,50], although higher temperatures provide greater activation energy, speeding up response and recovery times, and thus promoting desorption, they reduce the overall gas response.

### 4.2. Effect of NH_3_ Concentration

**Figure 12:**
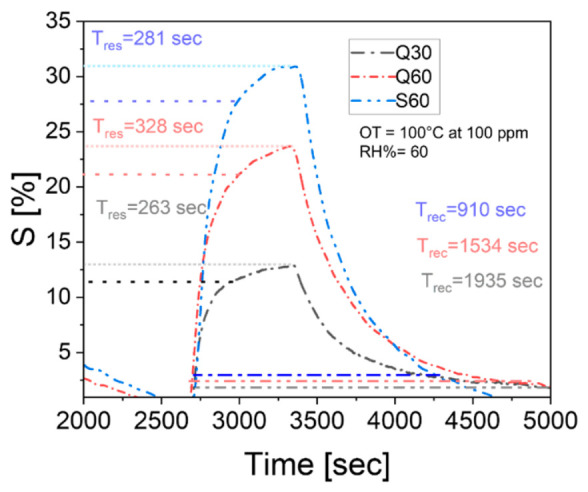
Response and recovery characteristics of WS_2_ sensing films to 100 ppm NH_3_ at 100 °C.

An increase in NH_3_ concentration (25–200 ppm) led to a corresponding increase in gas response. At low concentrations, only the number of adsorption sites contribute to the response, making differences between samples more pronounced. At higher concentrations, the response tends to approach saturation as the available active sites become occupied, reducing the relative differences between samples. Many studies have demonstrated a linear or near-linear relationship between concentration (usually in the range of 20–500 ppm) and gas response, allowing for accurate quantitative measurement [39,51]. This finding is also confirmed by our measurements of NH_3_ concentrations in the range of 50 to 200 ppm for all three WS_2_ samples studied at the optimal working temperature of 100 °C. These higher concentrations of NH_3_ may affect the response and recovery time of the sensor, as NH_3_ molecules have a strong binding to the WS_2_ surface, which may lead to a longer time required for complete desorption (recovery time) after exposure to high concentrations. As we can see from Figure 12, under the optimal parameters identified by us (operation temperature 100 °C, NH_3_ concentration 100 ppm), the shortest response times were 263 s and 281 s for samples Q30 and S60. In contrast, the longest response time, 328 s, was measured from the Q60 sample. The recorded recovery times ranged from 910 to 1935 s. 

### 4.3. Comparison with the Literature

Several high responsiveness and sensitivity WS_2_ sensors have been reported, but an enormous recovery time was often observed [50,52]. We found that Hau et al. (2026) achieved a sensor response of 528%, a response time of 105 s, a recovery time of 120 s at 50 °C and an NH_3_ concentration of 50 ppm for exfoliated WS_2_ nanosheets, which are better parameters but would not be stable for the long term due to chemical and mechanical instability [31]. In contrast, for WS_2_ films prepared using a similar technology as described in this paper (W film deposition and subsequent sulfurization), gas response was 2.8% at room temperature [39] and an NH_3_ concentration of 100 ppm, which is lower than the value of 13.8% achieved in our case at room temperature and a concentration of 100 ppm for sample Q60. However, a sensitivity of 0.2 ppm^−1^ was achieved at room temperature for Q60, with a moderate limit of detection of 3 ppm, primarily constrained by noise. (see Table 5).

## 5. Conclusions

This study investigated the preparation of suitable WS_2_ thin films for gas sensing applications to achieve high sensitivity at low operating temperatures. Gas sensing thin films were deposited by magnetron sputtering from a WS_2_ composite target and sulfurized at 800 °C for 30 and 60 min on quartz and sapphire substrates. Raman spectroscopy confirmed multilayer WS_2_ formation, with characteristic modes at 349 and 417 cm^−1^. XRD diffraction patterns of the sulfurized films showed the formation of the hexagonal 2H-WS_2_ phase on both substrates, with no detectable secondary phases, indicating that post-deposition sulfurization effectively restores a near-stoichiometric WS_2_ composition. The significant differences in surface morphology depending on the sulfurization duration and substrate type were revealed. It was found that crystallization on a sapphire substrate proceeds more slowly than on a quartz substrate. The WS_2_ films exhibited stable sensing performance at low operating temperatures from 30 to 150 °C and high sensitivity toward NH_3_ in the 10–200 ppm range. The highest value of sensor response of 45.5% was reached at an optimal operation temperature of 100 °C at 200 sccm NH_3_ for sample S60 on sapphire. A minimum value of the limit of detection of 0.6 ppm was achieved for the Q60 sample at an operating temperature of 150 °C, with WS_2_/quartz substrates affording greater sensing stability and reproducibility at near room temperature compared to WS_2_/sapphire.

## Figures and Tables

**Table 1:** Basic description of the four samples of WS_2_.

Identification of WS_2_	Substrate Type	Sulfurization Temperature	Sulfurization Time
Q30	Quartz	800 °C	30 min
Q60	Quartz	800 °C	60 min
S30	Sapphire	800 °C	30 min
S60	Sapphire	800 °C	60 min

**Table 2:** Calculated values of morphological characteristics of WS_2_ films.

Identification of WS_2_	Nanoparticles Coverage Area	Average Flake Thickness
Q30	12%	22 nm
Q60	23%	17 nm
S30	14%	X
S60	20%	18 nm

**Table 3:** The value of FWHM, separation, and intensity ratio of the A_1g_ and E^1^_2g_ Raman bands.

Identification of WS_2_	A_1g_ (cm^−1^)	FWHM (cm^−1^)	E^1^_2g_ (cm^−1^)	FWHM (cm^−1^)	A_1g_-E^1^_2g_ (cm^−1^)	A_1g_/E^1^_2g_
Q30	417	6.4	349.8	8.1	67.2	1.50
Q60	415.5	7.5	348.8	7.3	66.7	1.48
S30	417.7	5.1	349.9	7.9	67.8	1.60
S60	417.6	5.7	350.4	7.6	67.2	1.36

**Table 4:** Structural parameters of WS_2_ films from XRD analysis.

Identification of WS_2_	Q30		Q60	
Diffraction plane	002	004	002	004
2θ (WS_2_-PDF-08-0237)	14.32	28.88	14.22	28.88
2θ	14.21	28.66	14.18	28.70
FWHM	0.67	0.71	0.62	0.84
Crystallite size (nm)	13	12	14	10
**Identification of WS_2_**	**S30**		**S60**	
Diffraction plane	002	004	002	004
2θ	14.24	28.74	14.20	28.69
FWHM	0.56	0.69	0.40	0.49
Crystallite size (nm)	14	12	20	17

**Table 5:** Comparison of sensing parameters towards NH_3_ for pristine WS_2_ at RT.

Ref.	Morphology	Synthesis	Concentration Range & OT	Response (%)	Sensitivity (ppm^−1^)	LOD
Järvinen et al. (2019) [14]	WS_2_ (30 nm) SiO_2_/Si	W sputtered‚ sulfurized; Ti/Pt electrodes	10 ppm (RT)	~33%	0.10	~1 ppm
Fedorenko et al. (2025) [39]	WS_2_ on SiO_2_/Si ~30 nm	W sputtered then sulfurized	100 ppm (RT)	~2.8%	-	-
Y. Zhou et al. (2024) [52]	WS_2_ multilayer/sapphire	CVD growth Ti/Au IDE	100 ppm at 27 °C	10%	-	-
This work	Q60	WS_2_ sputtered then sulfurized	100 ppm	13.8%	0.20	~3 ppm

## Data Availability

The original contributions presented in this study are included in the article. Further inquiries can be directed to the corresponding author.

## Abbreviations

The following abbreviations are used in this manuscript:

2LA(M)	Second-order longitudinal acoustic phonon at the M-point Brillouin zone edge
FESEM	Field effect scanning electron microscope
FWHM	Full width at half-maximum
LOD	Limit of detection
min	Minutes
MOX	Metal oxide
OT	Operational temperature
ppm	Parts per million
ppb	Parts per billion
Q30	WS_2_ film on quartz substrate sulfurized for 30 min
Q60	WS_2_ film on quartz substrate sulfurized for 60 min
quartz	Quartz glass
RH	Relative humidity
RT	Room temperature
S	Response percentage
S30	WS_2_ film on a sapphire substrate, sulfurized for 30 min
S60	WS_2_ film on a sapphire substrate, sulfurized for 60 min
sccm	Standard cubic centimeters per minute
SEM	Scanning electron microscopy
TMDs	Transition metal dichalcogenides
XRD	X-ray diffraction
